# Toward precision longevity: aging interventions in the single-cell atlas era

**DOI:** 10.3389/fragi.2025.1674112

**Published:** 2025-12-05

**Authors:** Jason B. Chen, Miranda C. Wang, Shangyu Gong, Hongjie Li

**Affiliations:** 1 Huffington Center on Aging, Baylor College of Medicine, Houston, TX, United States; 2 Department of Molecular and Human Genetics, Baylor College of Medicine, Houston, TX, United States

**Keywords:** aging, longevity, anti-aging, pro-longevity interventions, single-cell atlas

## Abstract

With growing global interest in extending not only lifespan but also healthspan, healthy aging has emerged as a central goal in biomedical research. This review provides an overview of current longevity interventions, including genetic manipulations, dietary restriction, exercise, pharmacological treatments, targeting senescence and cellular reprogramming strategies, as studied in key model organisms such as *Caenorhabditis elegans*, *Drosophila melanogaster*, and *Mus musculus*. We examine the limitations and challenges associated with these approaches, particularly their variability across tissues and cell types. Furthermore, we emphasize the critical role of single-cell aging atlas technologies in uncovering cell-type–specific aging patterns and molecular signatures. By integrating single-cell data, we propose that future interventions can be more precisely designed to target aging at the cellular level, thereby enhancing the efficacy and specificity of longevity strategies.

## Introduction

As the global population ages, research on longevity has become increasingly important, with two-thirds of deaths worldwide resulting from age-related diseases. Interventions and strategies to facilitate healthy aging are a critical priority for the biomedical field (Grey, 2007). Treatments so far have mostly focused on the general whole-organism level, attempting to treat the entire body through the use of genetic regulation or drugs (Fontana et al., 2010; Jing et al., 2025).

However, in recent years, more emphasis has been placed on the specificity of cellular heterogeneity effects. The development of single-cell sequencing has enabled the analysis of tissues on a cell-by-cell basis, allowing for the differentiation between specific types of cells and the identification of their differences (Almanzar et al., 2020). Using this technology, aging cell atlases of several model organisms have been created, allowing for the study of aging at the cellular resolution, which is critical for developing precise longevity treatments (Zakar-Polyák et al., 2024).

**FIGURE 1:**
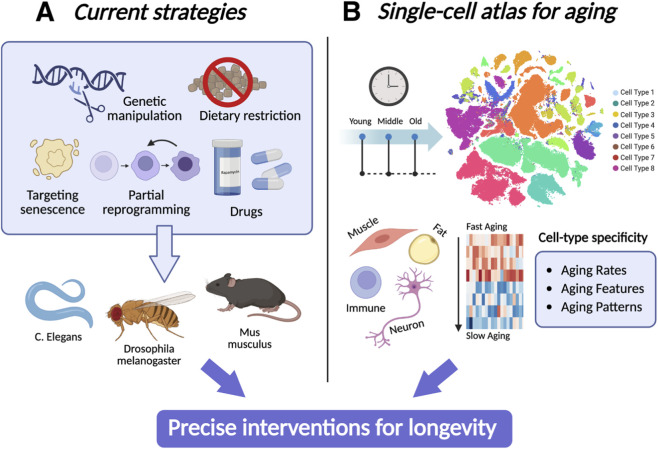
Strategies for Developing Precise Longevity Interventions. **(A)** The current anti-aging strategies include genetic manipulation, dietary restriction, the targeting of senescent cells, partial reprogramming, and pharmacological drugs, which are typically studied in model organisms in *Caenorhabditis elegans*, *Drosophila melanogaster*, and *Mus musculus*. **(B)** Aging Cell Atlas involves analyzing individual cells from organisms at different stages of life (young, middle, old).

In this review, we will briefly summarize current strategies for delaying aging and increasing healthspan, as well as their limitations. We will then discuss the findings of single-cell sequencing research and the construction of aging cell atlases. On this basis, we will emphasize the potential of applying single-cell atlas findings with conventional treatments to create precise aging interventions and longevity strategies (Figure 1).

### Current key strategies for aging interventions

Currently, many intervention strategies have been shown to increase lifespan and healthspan, ranging from dietary restriction, exercise, genetic manipulation, to pharmacologically-based treatments in model organisms.

Genetic manipulation is one longevity strategy. Specifically, targeting specific pathways has been proven to be able to extend lifespan. One important pathway is the insulin/insulin-like growth factor signaling pathway. In *C. elegans*, suppressing the pathway through the reduction in the activity of the *daf-2* gene, along with an activation of the *daf-16* gene, has been shown to be able to double the lifespan of the organism (Kenyon et al., 1993). In humans, the *FOXO3* gene is a homolog of the *daf-16* gene and has been associated with longer lifespan and healthspan when single-nucleotide polymorphisms occur (Willcox et al., 2008). Another important pathway is the mechanistic target of Rapamycin (mTOR) pathway. By inhibiting specific portions of the pathway, such as mTOR kinase or S6 kinase, the lifespan of *C. elegans* can be extended (Zhu et al., 2019). Because the mTOR pathway reduces autophagy, a vital component of *C. elegans* survivability, inhibiting the pathway can also increase autophagy activity and increase the lifespan of the organism (Hansen et al., 2008). Besides genetic manipulation, direct inhibition of the mTOR signaling pathway (Harrison et al., 2009) by feeding animals with Rapamycin can increase the lifespan in both fruit flies (Bjedov et al., 2010) and mice (Gkioni et al., 2025). Targeting of the mTOR pathway in *Drosophila* through overexpression of *dTsc1*, *dTsc2*, or dominant-negative forms of *dTor* also increased mean lifespan (Kapahi et al., 2004).

Dietary restriction (DR) – a reduction in overall nutrient intake without malnutrition–is another well-established strategy that extends lifespan and delays the onset of age-related diseases across diverse species, including *yeast*, *C. elegans*, *Drosophila*, mouse, and *Rhesus monkey* (Longo et al., 1997; Mair and Dillin, 2008; Anderson, et al., 2009; Grandison et al., 2009; Mattison et al., 2017). The beneficial effects of DR are mediated through several nutrient-sensing pathways, including the Insulin/Insulin-like Growth Factor Signaling (IIS), AMP-activated Protein Kinase (AMPK), and mTOR pathways (Fontana, et al., 2010). Building upon DR, nutritional interventions using bioactive dietary compounds–often referred to as nutraceuticals–have emerged as DR mimetics. Compounds such as resveratrol and polyphenols mimic DR’s beneficial effects by activating regulators of metabolism and stress resistance, including Sirtuin 1 (SIRT1), AMPK, and PGC-1α (Baur et al., 2006; Hubbard and Sinclair, 2014). These compounds exhibit broad, multiorgan effects that enhance mitochondrial biogenesis, vascular function, and glucose metabolism (Howitz et al., 2003; López-Lluch and Navas, 2016).

In parallel, exercise represents a powerful non-pharmacological intervention that enhances healthspan and mitigates many hallmarks of aging. Regular physical activity improves cardiovascular and metabolic health, promotes neurogenesis, and reduces chronic inflammation (Ruegsegger and Booth, 2018). At the molecular level, exercise induces the secretion of myokines–muscle-derived factors such as irisin, IL-6, and BDNF—that exert endocrine effects across organs, including the brain, liver, and adipose tissue (Pedersen and Febbraio, 2012; Wrann et al., 2013). These factors act as exercise mimetics, influencing nutrient-sensing and stress-response pathways similar to those affected by DR and mTOR inhibition. For example, BDNF enhances neuronal plasticity and cognitive resilience, linking peripheral metabolic activity to brain health (Mattson, 2012).

Targeting senescent cells has also been shown to increase lifespan. This is achieved through the use of genetic treatments or the administration of a class of drugs known as senolytics. The use of transgene INK-ATTAC (INK-linked Apoptosis Through Targeted Activation of Caspase), designed to eliminate senescent cells in mouse tissue, caused an increase in longevity and delays in age-related pathologies to be observed (Baker et al., 2011). Such an effect was also induced through the use of senolytic drugs such as dasatinib and quercetin, which were shown to increase tissue fitness in the limbs and improve cardiac health in mice (Zhu et al., 2015). For humans, administration of the senolytic drugs quercetin and dasatinib in those with Idiopathic pulmonary fibrosis, a cellular senescence disease, was correlated with increased physical function, such as walking distance and walking speed (Justice et al., 2019).

*In vivo* partial reprogramming is another promising and novel longevity intervention. This has been accomplished through the injection of Yamanaka factors, which rewind the cellular epigenetic clock without completely reverting cells into pluripotent stem cells. In mice, the introduction of transcription factors OCT4, SOX2, and KLF4 has been shown to induce epigenetic reprogramming and promote regeneration in retinal cells (Lu et al., 2020). Such factors were also shown to mitigate symptoms of aging, prolonging lifespan in progeroid mice and increasing tissue fitness in older mice (Ocampo et al., 2016). A similar effect was observed when human cells were treated to overexpress Yamanaka factors. Treatment with the mRNAs expressing *OCT4*, *SOX2*, *KLF4*, *c-MYC*, *LIN28*, and *NANOG* has been shown to induce a transient reprogramming of human cells (Sarkar et al., 2020). The treatment was found to reverse DNA methylation age, clear degraded biomolecules, and increase mitochondrial activity in fibroblasts and endothelial cells (Sarkar et al., 2020; Browder et al., 2022).

Blood-based rejuvenation treatments, including parabiosis (exposing old animals to young blood) and plasma exchange have shown that young or “reset” plasma can induce rejuvenation in organs such as the brain, muscle, liver, and bone by modifying the systemic signaling environment (Conboy et al., 2005; Villeda et al., 2011; Villeda et al., 2014). The beneficial effects of blood-based interventions are not attributable to a single molecular pathway or cell type. While targeting senescent cells and inflammatory cytokines shows promise, a diverse array of blood-borne proteins and signaling molecules, such as GDF11 (Loffredo et al., 2013), TIMP2, oxytocin, osteocalcin, and others, has been identified for contributing to tissue regeneration, reduced fibrosis, and improved cognitive or metabolic functions (Bitto and Kaeberlein, 2014; Castellano, 2019; Jeon et al., 2022).

### Translating longevity interventions into humans

A critical goal of aging research is to translate the profound lifespan and healthspan benefits observed in model organisms into safe and effective human therapies. This has led to the clinical investigation of several promising compounds, often repurposed drugs, as potential geroprotectors, agents that target the fundamental biology of aging (Table 1).

**TABLE 1:** Translational status of major longevity interventions.

Intervention strategy	*C. elegans* (Worm)	*D. melanogaster* (Fly)	*M. musculus* (Mouse)	Human studies
Genetic manipulation	Mutations in the *daf-2* gene, an insulin/IGF-1 receptor homolog, robustly double lifespan. (Kenyon et al., 1993)	Mutations in the insulin receptor substrate homolog chico extend lifespan significantly. (Clancy et al., 2001)	Mutations that reduce growth hormone signaling, as seen in Ames dwarf mice, lead to lifespan extension. (Brown-Borg et al., 1996)	Correlational evidence. Studies of long-lived cohorts have identified protective variants in homologous genes (e.g., *FOXO3*). (Willcox et al., 2008)
Dietary restriction (DR)	DR robustly extends lifespan and is a foundational model for studying nutrient-sensing pathways. (Lakowski and Hekimi, 1998)	Reduced nutrient intake extends lifespan, providing a key model for dissecting the roles of specific macronutrients. (Piper and Partridge, 2007)	DR is the most potent non-genetic intervention, extending lifespan and delaying age-related diseases in multiple strains. (Weindruch et al., 1986)	Healthspan benefits shown. The CALERIE trial confirmed metabolic and molecular benefits consistent with slower aging. (Belsky et al., 2023)
Pharmacological agents	Several compounds, including metformin, have been shown to extend lifespan, often by mimicking DR. (Onken and Driscoll, 2010)	Lifespan extension has been achieved with drugs like rapamycin, confirming the conservation of the mTOR signaling pathway’s role in aging. (Bjedov et al., 2010)	The NIA ITP has validated several drugs, most notably rapamycin and acarbose, for extending lifespan in genetically diverse mice. (Harrison et al., 2009), and trametinib and rapamycin combine additively to extend mouse healthspan and lifespan (Gkioni et al., 2025)	Clinical trials active. Rapamycin analogs (rapalogs) improve immune responses in the elderly, a key healthspan metric. (Mannick et al., 2014)
Senolytics	Not applicable. While cellular senescence markers exist, senolytic strategies are primarily studied in vertebrates	Not applicable. As with worms, this concept is not a primary research focus for aging in this model	Genetic clearance or pharmacological removal of senescent cells (senolytics) delays age-related pathology and extends healthspan. (Baker et al., 2011)	Early-phase clinical trials. Senolytics have shown potential in reducing senescent cell burden and improving function in age-related diseases. (Hickson et al., 2019)
Cellular reprogramming	Not applicable. This technology is specific to vertebrate cells and has not been applied to invertebrate aging models	Not applicable. The technique has not been adapted for this invertebrate model system	*In vivo* partial reprogramming using Yamanaka factors can reverse epigenetic age and ameliorate age-related decline. (Ocampo et al., 2016; Xu et al., 2024)	Not applied for systemic aging. Application is limited to *ex vivo* cell-based therapies for specific diseases in regenerative medicine

One of the most prominent examples is metformin, a first-line treatment for type 2 diabetes. In early studies with specific model organisms, metformin was shown to extend lifespan, likely by activating the AMP-activated protein kinase (AMPK) pathway, a key cellular energy sensor. However, these effects were not reproduced in the more rigorous studies of genetically heterogeneous mice conducted by the NIA Interventions Testing Program, underscoring the challenges of translating geroprotective benefits across diverse genetic backgrounds. Nevertheless, clinical investigations, such as the Metformin in Longevity Study (MILES, NCT02432287), have shown that metformin can still induce transcriptional changes in muscle and adipose tissue that are consistent with a younger biological profile. These foundational studies helped pave the way for the larger targeting aging with Metformin trial, a landmark study aiming to prove that a drug can target aging itself as a preventable condition, thereby delaying a cluster of age-related diseases.

Similarly, rapamycin, which extends lifespan across multiple species by inhibiting the mTOR pathway, has moved into human clinical trials. Given its potent immunosuppressive effects, clinical development has focused on derivatives (rapalogs) and intermittent dosing strategies to balance efficacy with safety. The Participatory Evaluation of Aging with Rapamycin for Longevity (PEARL) trial (NCT04488601), for example, is a pragmatic study designed to test whether intermittent rapamycin can modulate biomarkers of aging in healthy older adults.

Other compounds like acarbose, an alpha-glucosidase inhibitor that extends lifespan in mice, are also being investigated (NCT02953093). The successful progression of these drugs from animal models into human clinical trials demonstrates that the core aging pathways, such as nutrient sensing, are evolutionarily conserved and pharmacologically targetable. However, even these systemic interventions can have variable effects and side effects across different tissues, reinforcing the need for the more targeted, cell-type-specific approaches that single-cell atlases can help design.

### Limitations on current strategies

Although current healthy aging and longevity strategies offer numerous benefits, each also has its drawbacks. The interventions can benefit some cell types, but may also have detrimental effects on other types. For example, pharmacological interventions such as rapamycin, which extend lifespan by inhibiting the mTOR pathway, have been shown to impair spermatogenesis in both mice and humans (Zhu et al., 2019; Deutsch, et al., 2007). Similarly, genetic manipulation of the insulin/IGF-1 (IIS) pathway can promote longevity, but frequently results in reduced growth, fertility, or metabolic health due to the highly pleiotropic nature of these genes (Tatar et al., 2001; Kenyon, 2005). Another example is *in vivo* reprogramming. Although *in vivo* reprogramming was shown to reverse aging in fibroblasts and endothelial cells, continuous administration of Yamanaka factors has also been proven to result in hepatic and intestinal failure, resulting in death (Parras et al., 2023). Overall, these drawbacks underscore the need for a more comprehensive analysis of cellular heterogeneity to identify precise interventions for healthy aging.

### Single-cell atlas in model organisms uncovers novel, cell-type–specific insights into the aging process

The high-throughput, whole organism single-cell sequencing is emerging as a critical method for aging research. Unlike traditional bulk sequencing methods which average out molecular signals, single-cell analysis provides the resolution needed to observe the distinct aging speeds of different cells across tissues (Li, 2021; Cohn et al., 2023). This allows us to move beyond broad observations to a more precise, cellular-level understanding of aging. By creating aging and age-related disease cell atlases, comprehensive maps comparing the gene expression profiles of young and old individuals. For example, the researchers can pinpoint the specific cell populations from the whole *Drosophila* organism that are most vulnerable in response to neurodegeneration conditions (Park et al., 2025). While considering species-specific physiological differences between worms, flies, mice, and humans, the identification of conserved aging trajectories of various genes and pathways will help researchers design more precise interventions. This cross-species approach is justified by the remarkable conservation of core longevity-regulating genes (e.g., *FoxO*) and molecular pathways (e.g., mTOR, Insulin/IGF-1 signaling) from invertebrates to humans, which provides a strong rationale for translating foundational discoveries into clinical strategies. This will help researchers design more precise interventions to combat aging and increase lifespan (Aldridge and Teichmann, 2020; Miao et al., 2021; Cohn et al., 2023).

#### *Caenorhabditis elegans* aging cell atlas

The creation of a worm cell atlas demonstrates aging trajectory differences among distinct cell types. The dataset profiled over 47,000 somatic cells. They found the expression of energy metabolism genes universally declined across all 211 identified cell clusters, while the stress-response signatures of specific cell types varied greatly (Roux et al., 2023). To identify novel regulators of aging, the authors used RNA interference to knock down transcription factors (TFs) whose expression changed with age but had not been previously linked to longevity. Three genes, *gei-3*, *lsy-2*, and *mef-2*, were found to accelerate aging phenotypes when suppressed, identifying them as potential new regulators of longevity. Notably, *gei-3* and *lsy-2* indicated a stronger activity in aging neurons, whereas *mef-2* was more active in the epithelium and the hypodermis, demonstrating cell-type-specific aging regulatory mechanisms.

Neurons experienced the greatest transcriptomic change with age, indicating that they may undergo earlier and more rapid aging in *C. elegans.* This notion was supported by another aging atlas study, Cell Atlas of Worm Aging (Gao et al., 2024). For example, cytosolic chaperones were upregulated in subsets of neurons as a response to proteotoxic stress. Meanwhile, ER stress response genes were broadly downregulated in neurons during aging, highlighting heterogeneous responses even within the same cell type. Compared to other tissues, such as muscle or hypodermis, which aged more uniformly through consistent downregulation of energy metabolism and mitochondrial genes, neuronal aging appeared more variable. This is reinforced by findings from the new study, which supports a key insight from the *C. elegans* atlas is that neurons are among the earliest and most variably aging cell types, experiencing significant transcriptomic changes before tissues like muscle. This suggests that the nervous system may be a critical driver of the initial stages of organismal aging in worms. Furthermore, the atlas was instrumental in identifying new longevity regulators (*gei-3*, *lsy-2*, *mef-2*) and demonstrating that they operate in a cell-type-specific manner. This provides a direct rationale for developing therapies that target specific cell populations, such as neurons, to delay the early onset of aging (Gao et al., 2024).

Additionally, understanding this development has enabled the creation of tissue aging clocks using machine learning methods to assess anti-aging interventions. Suppression of the insulin/IGF-1 pathway (*daf-2*) and mTOR pathway (*rsks-1*) was associated with slowed aging in specific tissues such as the hypodermis, muscle, and intestine, demonstrating the potential of single-cell atlases as platforms for evaluating future anti-aging treatments (Gao et al., 2024).

#### *Drosophila* aging cell atlas

The Aging Fly Cell Atlas (AFCA) was recently created for mapping the cellular changes of the whole organism of *Drosophila* at different ages (Lu et al., 2023). The study found the greatest decrease of cell number in muscle cells, most notably in indirect flight, visceral, and skeletal muscle, among all cell types. This observation aligns with sarcopenia, the age-related loss of muscle mass and strength, a conserved aging phenotype observed across mammals, including humans. In contrast, most neuronal cell types showed minimal cellular composition change with age.

To quantify biological aging, regression-based aging clocks were trained to predict the biological age for each cell type, denoting high average performance. Four different aging features were also used to rank cell types, with a higher rank indicating a higher aging rate: cell composition changes, differentially expressed genes (DEGs), change of expressed gene numbers, and cell identity decline. Out of 163 distinct cell types, the top three fastest aging were all surprisingly discovered to be adipose cell types: oenocytes, fat body cells, and pericerebral adult fat mass—suggesting that those cells age faster than other cell types (Lu et al., 2023). Following them in the ranking were male accessory gland main cells, indirect flight muscle, and enteroblast. Generally, neurons and glia from the nervous system age more slowly than other cell types.

The AFCA provides a critical insight by disentangling structural decline from molecular aging trajectories. Muscle tissues showed the most pronounced loss in cell number, highlighting a clear structural phenotype, whereas adipose tissues such as oenocytes and the fat body exhibited the earliest and most accelerated molecular aging signatures. This contrast indicates that efforts to preserve muscle integrity require fundamentally different approaches from those needed to counteract the metabolic and inflammatory dysfunction of aging adipose tissue. Collectively, these findings position adipose tissue as a central hub for early molecular intervention to slow down systemic aging through tissue-tissue communication, and future functional studies are required to test this hypothesis.

#### Mouse aging cell atlas

The single-nucleus transcriptome mouse atlas PanSci identifies over 300 distinct cell types covering 14 different tissues or organs across five life stages in both male and female mice (Zhang et al., 2024). PanSci provides a comprehensive catalog of aging-related cell population changes in the mouse. During early adulthood, from 3 to 12 months, cellular depletion is most prominent in adipose, muscle, and epithelial lineages. In contrast, later adulthood, from 12 to 23 months, reflected a substantial increase in various immune cells. These findings underscore the benefits of initiating anti-aging interventions at an early stage.

Additionally, the study found both general and organ-specific changes within broadly distributed cell types. Early immune expansion occurred predominantly in the kidney and lung, whereas later expansions were observed in the liver and adipose tissues, potentially indicating the onset of organ-specific aging diseases. Likewise, the consistent decrease in CD4^+^ naive T cells and increase in CD8^+^ cytotoxic T cells indicate a universal regulatory mechanism across the entire body (Zhang et al., 2024).

Another Mouse Aging Cell Atlas, called *Tabula Muris Senis*, supports the notion of distinct cell-type-specific aging trajectories (Almanzar et al., 2020). For instance, they found that age-related decline of T cell populations, which is associated with increased risk of infectious disease and cancer, may also occur in the spleen and the mammary gland. Additionally, scRNA-seq enabled the reconstruction of B cell and T cell receptors, revealing an increase in clonality from 3-month to 24-month-old mice, indicating a decrease in unique immune cells. This finding explains how aging often contributes to a weaker immune system due to reduced adaptability of older individuals to new pathogens. Trajectory analysis also suggests that microglial cells in the aging brain resemble those of Alzheimer’s, with 55 out of the top 200 differentially expressed genes shared between both lists, linking normal aging to potential disease pathways.

The mouse aging atlases provide compelling evidence that immune system dysfunction is a central driver of organismal aging. Coordinated body-wide changes, including the depletion of naïve T cells and the expansion of cytotoxic and pro-inflammatory immune populations, help explain the emergence of chronic low-grade inflammation, or “inflammaging”, together with the progressive decline in immune competence. Equally important, the atlases link molecular hallmarks of normal aging directly to age-related disease pathways. For instance, aging microglia progressively adopt transcriptional states resembling those associated with Alzheimer’s disease. These findings establish a strong mechanistic rationale for targeting specific immune cell populations as a strategy to mitigate multiple age-related pathologies in parallel.

Although recent single-cell atlases of aging initially appear to offer conflicting perspectives on the primary drivers of aging, these differences highlight a critical principle: aging is a complex, multisystem process in which species-specific physiology dictates which tissues are most vulnerable. Each of these pioneering studies, conducted independently, has emphasized the most obvious features within its respective model organism, and their interpretive scope is further constrained by the current scarcity of comparable human single-cell aging atlases. Nonetheless, emerging cross-species comparisons are beginning to reveal conserved signatures. For instance, the *Drosophila* atlas identified age-associated downregulation of ribosomal protein genes as a key molecular clock feature, a pattern consistent with findings in human peripheral blood cells further supported by evidence linking impaired protein synthesis to chronic inflammation (“inflammaging”) (Zhu et al., 2023). Taken together, these observations underscore two important implications for human longevity: first, different tissues may act as dominant drivers of aging depending on context; and second, integration across diverse models can uncover evolutionarily conserved pathways, such as the decline of protein synthesis machinery that constitute high-priority targets for broadly effective aging inventions.

### Integrating model organism insights, single-cell atlases for precise aging intervention

The combination of single-cell atlas data, findings from model organisms, and advanced drug delivery technologies are transforming aging interventions (Muñoz-Espín et al., 2018; Ezike et al., 2023). Model organism studies have shown that dietary restriction, rapamycin, and metformin can target conserved aging pathways (Mair et al., 2003; Bjedov et al., 2010; López-Lluch and Navas, 2016; Mattison et al., 2017; Kulkarni et al., 2020; Gkioni et al., 2025), while single-cell atlases identify the most affected cell types. This offers an opportunity to understand how broad systemic interventions such as DR, nutraceuticals, and exercise influence distinct cellular states and tissue microenvironments during aging. Single-cell atlases of aging reveal that cellular responses to metabolic and signaling interventions are highly heterogeneous across tissues, suggesting that global interventions engage both shared and cell type–specific protective mechanisms. These strategies thus provide a bridge between organism-wide regulation and cell-type precision, complementing targeted genetic or pharmacological approaches. Integrating these insights with single-cell atlases will advance our mechanistic understanding of how pleiotropic interventions modulate cellular aging trajectories across the body. As single cell atlases grow to cover more human tissues, they will help pinpoint key molecular drivers of aging and guide the adaptation of effective interventions from animals to humans.

Single-cell atlases enable tailored, multi-target strategies for complex aging pathways within specific cell types. This approach is conceptually similar to modern cancer combination therapies, where drugs targeting cell proliferation are paired with agents that stimulate an anti-tumor immune response. In the context of aging adipocytes, where single-cell data reveals both increased inflammation and dysregulated nutrient sensing (mTOR), these distinct issues (mTOR) (Cai et al., 2016; Fernandes and Demetriades, 2021) could be tackled simultaneously. For example, one could use mRNA-LNPs (lipid nanoparticles) to deliver anti-inflammatory agents like IL-10 and separate LNPs to deliver mTOR inhibitors (Reyes-Esteves et al., 2025; Xu and Xia, 2023; Zong et al., 2023; Alharbi et al., 2024). This represents a crucial shift from the current 'one-size-fits-all' model, where a single geroprotector is expected to broadly combat the multifaceted nature of aging.

The diversity of aging across cell types demands customized therapies. For instance, single-cell atlases reveal that in aged skin, keratinocytes are a primary source of senescence-associated inflammation (Zou et al., 2021). Therefore, a rational approach is to use ligand-modified nanoparticles (liposomes) to deliver senolytics. In parallel, these atlases show that dermal fibroblasts primarily exhibit dysregulation of the Extracellular Matrix (ECM) and reduced collagen synthesis. Consequently, a complementary strategy would employ different delivery systems, such as viral vectors or other LNPs, to enhance collagen production in fibroblasts. Here, the choice of delivery technology is deliberately matched to the therapeutic goal; a LNP is ideal for the safe, transient delivery of a senolytic drug, whereas a viral vector is advantageous for its ability to provide the stable, long-term gene expression needed to rebuild the ECM. This illustrates how future interventions will not only target the right cell but will also use the optimal delivery system to achieve the desired biological outcome and duration of effect. Similarly, targeted delivery of IL-7 via exosomes to T-cell progenitors addresses age-related immune decline (Kim et al., 2006; Nguyen et al., 2017). By engineering delivery systems to target cell-type-specific markers, interventions can precisely address each population’s unique aging drivers.

Aging is also nonlinear, with molecular changes accelerating around ages 44 and 60 in humans, increasing disease risk (Shen et al., 2024). For instance, lipid metabolism pathways in endothelial cells could be targeted around age 44 using LNPs or gene editing to reduce cardiovascular risk, while immune interventions at age 60 could rejuvenate T cells. Integrating single-cell data with these temporal insights enables the delivery of precisely timed cell-specific treatments to optimize aging intervention outcomes.

## Conclusion

In summary, the field of longevity research is rapidly advancing beyond whole-organism interventions toward increasingly precise, cell-type–specific strategies. Breakthroughs in single-cell sequencing and the creation of detailed cellular atlases across multiple organisms have revealed the profound heterogeneity of aging between tissues and among individual cell types-insights that are now key to devising more effective and personalized anti-aging therapies. While interventions such as dietary restriction, genetic manipulation of key pathways, senolytics, *in vivo* reprogramming, and blood-based rejuvenation have demonstrated significant benefits in model organisms, their broad application in humans remains limited by variable effects across different cell populations and potential adverse outcomes. The integration of single-cell atlas data with advanced drug delivery methods-such as lipid nanoparticles for multi-tissue targeting, GalNAc conjugates for liver-specific therapies, and tailored platforms like ligand-modified nanoparticles and exosomes-holds transformative potential. These approaches enable the translation of robust, conserved molecular findings from animal models directly into targeted human therapies. As aging atlases expand to encompass more and more human tissues and standardized cross-species comparisons become possible, we anticipate a future where interventions inspired by model organisms can be adapted with precision to humans. This convergence of cellular-resolution data and next-generation delivery technologies will empower the development of truly individualized, cell-type-specific geroprotective interventions, opening a promising new chapter in our quest to delay aging, extend healthspan, and reduce the burden of age-related disease.
